# The adult microbiome of healthy and otitis patients: Definition of the core healthy and diseased ear microbiomes

**DOI:** 10.1371/journal.pone.0262806

**Published:** 2022-01-24

**Authors:** Maria Burton, Janina A. Krumbeck, Guangxi Wu, Shuiquan Tang, Aishani Prem, Aditya K. Gupta, Thomas L. Dawson

**Affiliations:** 1 Zymo Research Corporation, Irvine, CA, United States of America; 2 Mediprobe Research Inc., London, ON, Canada and University of Toronto, Toronto, ON, Canada; 3 Skin Research Institute, Singapore, Singapore; 4 Dept of Drug Discovery, College of Pharmacy, Medical University of South Carolina, Charleston, SC, United States of America; University of Illinois Urbana-Champaign, UNITED STATES

## Abstract

Otitis media (OM) and externa (OE) are painful, recurrent ear conditions. As most otitis publications focus on the bacterial content of childhood ears, there remains a dearth of information regarding the adult ear microbiome including both bacteria and fungi. This study compares the outer ear microbiome of healthy adults to adults affected by OE and OM using both intergenic-transcribed-spacer (ITS) and 16S-rDNA sequencing. The adult ear core microbiome consists of the prokaryote *Cutibacterium acnes* and the eukaryotic *Malassezia arunalokei*, *M*. *globosa*, and *M*. *restricta*. The healthy ear mycobiome is dominated by *Malassezia* and can be divided into two groups, one dominated by *M*. *arunalokei*, the other by *M*. *restricta*. Microbiome diversity and biomass varied significantly between healthy and diseased ears, and analyses reveal the presence of a potential mutualistic, protective effect of *Malassezia* species and *C*. *acnes*. The healthy ear core microbiome includes the bacteria *Staphylococcus capitis* and *S*. *capitis/caprae*, while the diseased ear core is composed of known bacterial and fungal pathogens including *Aspergillus* sp., *Candida* sp., *Pseudomonas aeruginosa*, *S*. *aureus*, *and Corynebacterium jeikeium*. The data presented highlight the need for early detection of the cause of otitis to direct more appropriate, efficient treatments. This will improve patient outcomes and promote improved antimicrobial stewardship.

## Introduction

Acute and chronic ear infections are of considerable importance, among the most common reasons for physician visits and resulting antibiotic treatment [1–3]. Otitis was previously considered more prevalent in children under the age of 15 [4], but a recent study found that about half of physician visits for otitis complaints were from adults [2]. The most common ear infections include otitis media (OM) and otitis externa (OE). OM is characterized by middle ear inflammation with subtympanic fluid accumulation, caused or promoted by growth of bacterial, fungal, and/or viral pathogens leading to infection, swelling, redness, and pain [5]. OE (“swimmer’s ear”), consists of outer ear canal infection and inflammation accompanied by pain, redness, and tenderness with occasional purulent exudates. OE presents as either an acute (AOE) or chronic (COE) disease: AOE is believed to be of bacterial origin, while COE is more often caused by fungi. Treatments for OM and OE consist of oral antibiotics in at least 25% of patients [1]. OM and OE are often thought to result from a polymicrobial infection with biofilm formation leading to antibiotic resistance phenotypes, which may explain why at times antibiotic treatments provide limited benefit [4], as well as limited effectiveness due to the increase of resistant microbes or treatment with an incorrect antimicrobial.

Current diagnostic methods include culture-based techniques and molecular tests e.g., PCR-based microbial panels. However, less than 1% of all microbes can be cultured using standard methods [6]. While a majority of the microbes detected in this study, and the ear core microbiome, are able to be cultivated, they require diverse cultivation techniques and have vastly different growth characteristics. For example, the most common components for the mycobiome, and clear differentiators of health and disease, are *Malassezia* species. Most clinical testing laboratories minimally routinely test for fungi at all, and the fastidious *Malassezia* species *M*. *restricta* and *M*. *arunalokei* would be very likely to be under-reported. This is also the case for many *Cutibacterium* species, which require anaerobic conditions to grow in culture. Hence, culture techniques often lack the sensitivity and speed needed to detect the variety of pathogens that may be present in a polymicrobial ear infection, which include biofilms, and difficult to grow, anaerobic bacteria and/or fungal pathogens [7, 8]. Although more sensitive and faster, quantitative PCR-based microbial testing is limited to the number of microbial targets (usually 8–20) contained in the panel. These limitations of existing diagnostic methods challenge the accurate diagnosis and proper management of ear infections.

While the microbiology of adult OM remains poorly investigated, culture methods have implicated otopathogens such as *Haemophilus influenzae*, *Streptococcus pneumoniae*, and *Moraxella catarrhalis* as primary bacterial causes of childhood acute OM (AOM) [9]. 16S Next-Generation DNA Sequencing (NGS) has confirmed these as primary otopathogens, but also added *Turicella otitidis*, *Alloiococcus otitidis*, and *Staphylococcus auricularis* [10], highlighting that NGS testing has the potential to provide more detailed information about the bacterial etiology of AOM and has been instrumental in the systematic detection of novel candidate organisms [11]. *A*. *otitidis* remains controversial as a pathogen as it is found in healthy ears, highlighting the need to further NGS-based analysis to understand how microbial species can be, at times, either pathogenic or benign. The OE microbiome is even less well reported, with *Staphylococcus aureus*, *Staphylococcus haemolyticus*, *Klebsiella*, and *Рseudomonas аеruginosа* as primary bacterial pathogens [9, 12]. However, these bacterial culture and DNA sequencing-based approaches are limited to bacterial detection and cannot properly diagnose the 5–10% of OE cases potentially caused by fungi. These fungal OE cases will not respond to antibacterial treatments, lead to excessive antibiotic use, prolonged symptoms, and wasted medical expenditure.

NGS has the potential to impact patient care by improving clinical diagnostic sensitivity and enabling targeted treatments through precise, unbiased, and comprehensive species-level microbial identification of bacteria and fungi, with the ability to generate semi-quantitative data [7, 13, 14]. The aim of this study was to use an NGS-based microbial test for the diagnosis of OM and OE in an adult population. Microbial profiles of OM and OE individuals were compared to a healthy cohort to investigate how the ear microbiome is altered under these conditions and to investigate the possibility of microbial perturbation or pathogen overgrowth in otitis. To date, the vast majority of sequence and culture-based investigations of OM and OE have focused on children and included only bacterial detection methods [15–17]. This study highlights how NGS can provide novel insights into the adult otitis microbiome including its role in health and disease. Accurate, fast, and early detection of the causes of OE and OM can guide more effective treatments, decrease the need for antibiotic prescriptions, promote good antibiotic stewardship, and greatly improve patient care and outcomes [18].

## Material and methods

### Study design and subject population

Healthy subjects as well as patients with different disorders of the ear were recruited from a private practice for enrollment in this study. This was a single-center study and specimens were collected from Tustin Ear, Nose & Throat Sinus and Allergy Center, practice of Dr. Charles Oh, a board-certified Otolaryngologist who performed all the diagnoses and sample collection. This study was approved by the Zymo Research Corporation Institutional Review Board (IRB) and all participating subjects provided a written informed consent prior to the start of the study. To determine the microbial composition of the ear, samples were collected from 46 healthy individuals with specimens taken from both ears, for 92 samples total, ages 22 to 60 years with no known history disorders of ear or sino-nasal cavities and no chronic medical conditions including asthma, bronchitis, or allergic rhinitis. Healthy participants were from the local Irvine, Tustin, CA, USA areas and data from these were compared to 22 patients with otitis externa (OE) and 48 patients with otitis media (OM) (Table 1). Exclusion criteria included patients receiving any antimicrobial treatment at time of collection and those with non-infectious disorders of the ear.

**Table 1:** Clinical characteristics of study subjects.

Characteristics	Healthy (*n* = 92)	Otitis (*n* = 70)
Male/Female	23/23 (bilateral)	35/37
Age (Median)	30	64
Age range	22–60 years	22–92 years
Otitis Media (OM)	—	48/70
Otitis Externa (OE)	—	22/70

### Sample collection

Samples were collected using the collection kit provided with the PrecisionBIOME NGS Microbial test (Pangea Laboratory, Tustin, CA, USA). This kit contains sterile oropharyngeal and nasopharyngeal swabs and collection tubes pre-filled with a nucleic acid-stabilizing solution called DNA/RNA Shield^TM^ (Zymo Research Corp., Irvine, CA). This solution can preserve the microbial DNA present in the specimen at room temperature for up to 30 days (S1 Fig). Both left and right ears from all healthy participants were swabbed using a sterile nasopharyngeal swab provided with the kit. Swabbing was performed in a standard way by rotating the swab at least five full turns inside the ear canal until swab was saturated. Right after swabbing, the swab was placed inside the collection tube and the rest of the swab stem was discarded at the breakpoint. Tubes were tightly capped and sent for 16S and ITS analysis through the PrecisionBIOME^TM^ NGS Microbial test. A total of 92 healthy ear specimens were collected and analyzed.

Specimens from adult subjects with different ear infections including OM and/or OE were collected using a similar procedure as described above. A total of seventy otitis patients (70) with a median age of 64 years and suffering from either OM or OE (Table 1) were recruited for microbial analysis and pathogen identification using the PrecisionBIOME^TM^ NGS Microbial test. For sample collection, an otoscope and microscope were used to identify the site of infection. For all OM cases, sampling was performed through the outer ear canal into the middle ear. Middle ear fluids were collected by aspiration (tympanocentesis) and placed directly inside the specimen collection tube. In cases of ruptured ear drums, fluids were collected with a flexible nasopharyngeal swab using an auditory speculum. For OE cases, the outer ear canal skin including ear exudates were collected using a sterile nasopharyngeal swab included in the collection kit. Otitis media was the most common type of ear infections analyzed in this study (N = 48/70).

### DNA extraction, library preparation, and sequencing

Shortly after collection, specimens were sent for analysis through the PrecisionBIOME™ NGS Microbial Test (Pangea Laboratory, Tustin, CA, USA). PrecisionBIOME™ is an NGS test that provides accurate and fast identification and quantification of both bacterial and fungal species present in a clinical specimen along with antibiotic susceptibility information. Briefly, microbial DNA from the specimen was extracted using the ZymoBIOMICS DNA Miniprep Kit according to the manufacturer’s instructions (Zymo Research Corporation, Irvine, CA). Extracted DNA was next prepared for NGS analysis following a workflow which included library preparation using the Quick-16S™ NGS library prep kit (Zymo Research Corporation, Irvine, CA), sequencing of barcoded amplicons with the MiSeq sequencing platform (Illumina, San Diego, CA), and bioinformatics analysis using a proprietary PrecisionBIOME™ bioinformatics pipeline capable of producing species-level resolution of bacterial and fungal sequences. Positive and negative controls (transport medium alone and unused swabs) were also included in the NGS workflow. To control for contamination cell and DNA mock communities were used as positive controls (ZymoBIOMICS microbial community standard, catalog Nos. D6300 and D6305; Zymo Research Corp) from the DNA extraction process. ZymoBIOMICS Microbial Community Standard (Zymo Research Corporation, Irvine, CA) was used as a positive control to monitor the performance of all steps of the NGS workflow including the bioinformatic analysis. Potential sequencing errors and chimeric sequences were also removed with the DADA2 pipeline. Absolute abundance of total bacteria and fungi was determined using the Femto Bacterial and Fungal DNA Quantification kits (Zymo Research Corporation, Irvine, CA) according to manufacturer’s protocols.

### Analysis of microbiota

Microbiota profiling was determined using the PrecisionBIOME™ bioinformatics analysis pipeline. Uclust was used to perform taxonomic classifications using a PrecisionBIOME™ custom proprietary database. Phylotypes were computed as percent proportions based on the total number of sequences in each sample. Relative abundances of bacteria compared to fungi were determined assuming an equivalency of one 16S rDNA copy to one fungal ITS copy. While it is unlikely that the number of 16S or ITS copies change in any given, this is an assumption, which may be considered a limitation of the analysis presented here. Absolute microbial quantification was achieved using a real-time PCR approach using primers targeting the V1-V3 and ITS regions for bacterial and fungal quantification, respectively. Species level resolution of this sequencing approach was previously confirmed by shotgun sequencing [19].

### Statistical analyses

Unless otherwise stated, results were expressed as average values with standard deviation. Measurements of α- diversity and evenness were calculated using the Shannon index and the number of observed species. β-diversity was calculated using Bray-Curtis distance at the species taxonomic level for both bacteria and fungi. Linear discriminant analysis (LDA) and effect size (LEfSe) were used to identify taxa that were significantly enriched in each group using the default settings (QIIME version 1.9.1, *P* value > 0.05 was considered significant) [20]. Analyses of variance and false discovery rate control to correct for type I errors were performed on the species-level relative abundance data of this analysis. Species with a P value < 0.05 were considered significant. A presence-absence data matrix of species by site was generated by assuming species with abundance greater than 1% as presence and less than 1% as absent. The “co-occur” function from R (“cooccur” package in R version 3.5.2, R Core Team, 2013) was used to generate pairwise classification of species having positive, negative, and random associations. The core microbiome was determined based on taxa detected with ≥ 5% relative abundance and in ≥ 50% of all samples. GraphPad Prism (version 8) software was used for data visualization.

## Results

To identify possible causative agents of adult ear infections, we compared the bacterial and fungal microbial profiles of three study groups: healthy, asymptomatic adults, otitis media (OM) patients, and otitis externa (OE) patients. NGS analysis detected both bacterial and fungal organisms, with a total of 877 different bacterial and 277 fungal species detected (S1 and S2 Tables). To measure similarities/differences in the microbial composition a β-diversity analysis showed a clear bacterial composition separation of healthy vs infected groups, with OE and OM specimens clustering. No clustering was seen for the fungal component (S2 Fig). As a further control for internal consistency, we compared the left and right samples from healthy ears and found no significant differences (S3 Fig).

### Bacteriome composition and diversity

**Fig 1:**
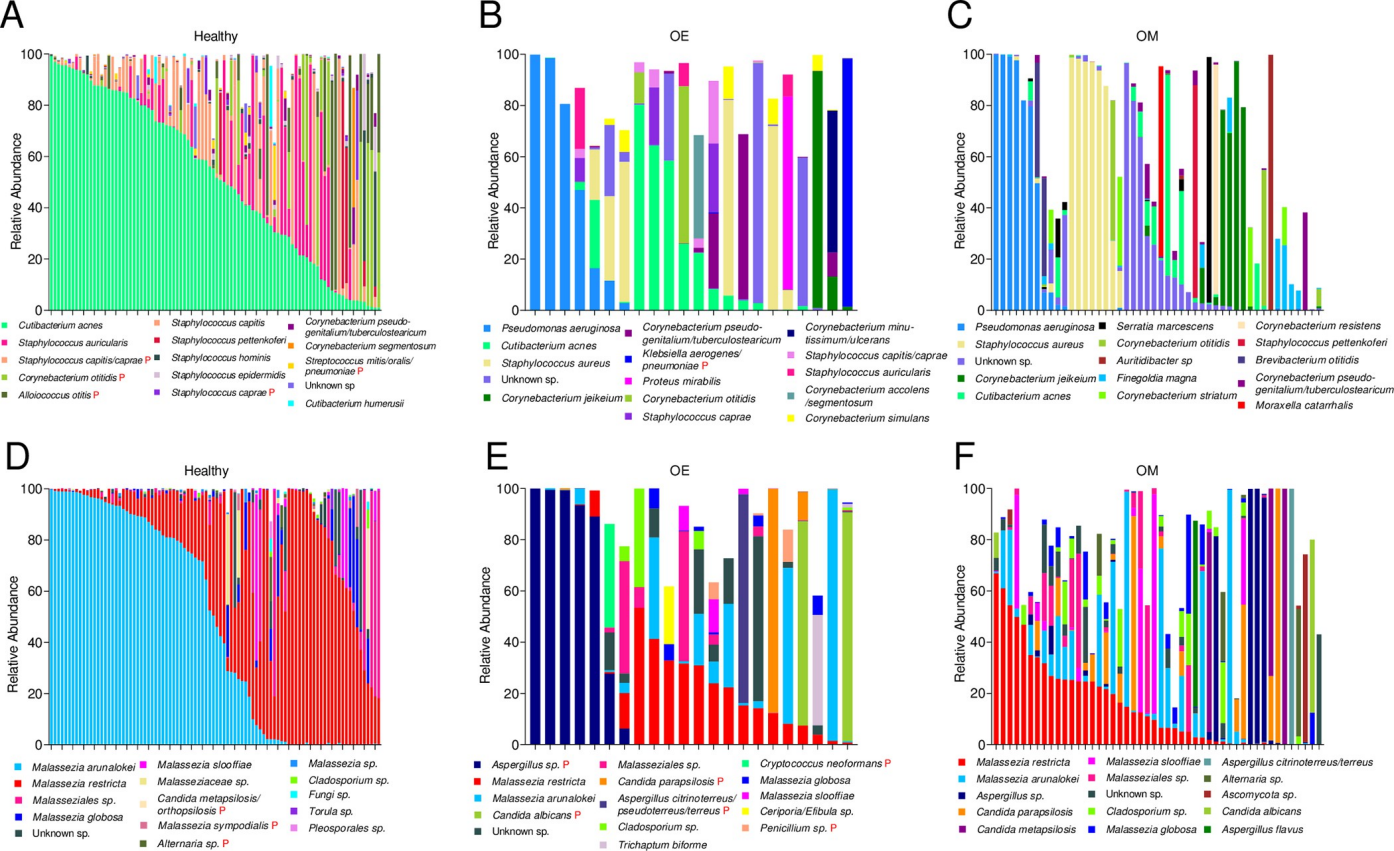
Microbiome profile of individual samples for bacteria (A-C) and fungi (D-F) for each group, healthy, OE (otitis externa), and OM (otitis media). Shown are the top 15 most abundant species based on relative abundance per group. A red letter (P) highlights those species known to be otitis pathogens. To ease comparison, bacterial and fungal species are consistently colored between graphs. Further information about the relative abundances of all detected species per individual can be found in S1 Table (bacteria) and S2 Table (fungi).

At the phylum level, the bacteriome of healthy ears was dominated by Firmicutes and Actinobacteria while in otitis ears Proteobacteria and Bacteroides were more often detected. At the species level *Cutibacterium acnes* dominated the healthy ear bacteriome with the highest average relative abundance (51.83%) and frequency (92/92 samples). The following top four bacteria detected in healthy ears were *S*. *auricularis* (10.07%, 34/92), *S*. *capitis/caprae* (9.47%, 82/92), *Corynebacterium otitidis* (6.67%, 28/92), and *A*. *otitis* (6.16%, 29/92). Interestingly, five species previously reported as potential pathogens were detected in the healthy group, although at lower abundances *(S*. *capitis/caprae*, *C*. *otitidis*, *A*. *otitis*, *S*. *caprae*, and *Streptococcus mitis/oralis/pneumoniae* (Fig 1A).

The Proteobacterium *P*. *aeruginosa* was the most abundant species on average in the OE group (16.23%, 12/22), followed by *C*. *acnes* (13.18%, 17/22), *S*. *aureus* (11.96%, 8/22), and *Corynebacterium jeikeium* (4.87%, 5/22) (Fig 1B). In total, eight previously defined otopathogens were detected amongst the top 15 most abundant species, with *P*. *aeruginosa*, *S*. *aureus*, *C*. *jeikeium*, *Klebsiella aerogenes/pneumoniae* (4.41%, 2/22), *Proteus mirabilis* (3.42%, 1/22), *C*. *otitidis* (3.35%, 4/22), and *S*. *caprae* (2.65%, 3/22) representing at least 2% of the average abundance.

The OM group had *P*. *aeruginosa* as the most abundant species (13.10%, 20/48), followed by *S*. *aureus* (12.94%, 14/48), *C*. *jeikeium* (7.17%, 11/48), and *C*. *acnes* (4.45%, 36/48) (Fig 1C). Ten previously defined otopathogens detected amongst the top 15 most abundant species. Of these, *P*. *aeruginosa*, *S*. *aureus*, *C*. *jeikeium*, *S*. *marcescens*, *C*. *otitidis* (2.43%, 9/48), and *Auritidibacter sp*. (2.11%, 3/48) represented at least 2% of the average relative abundance.

**Fig 2:**
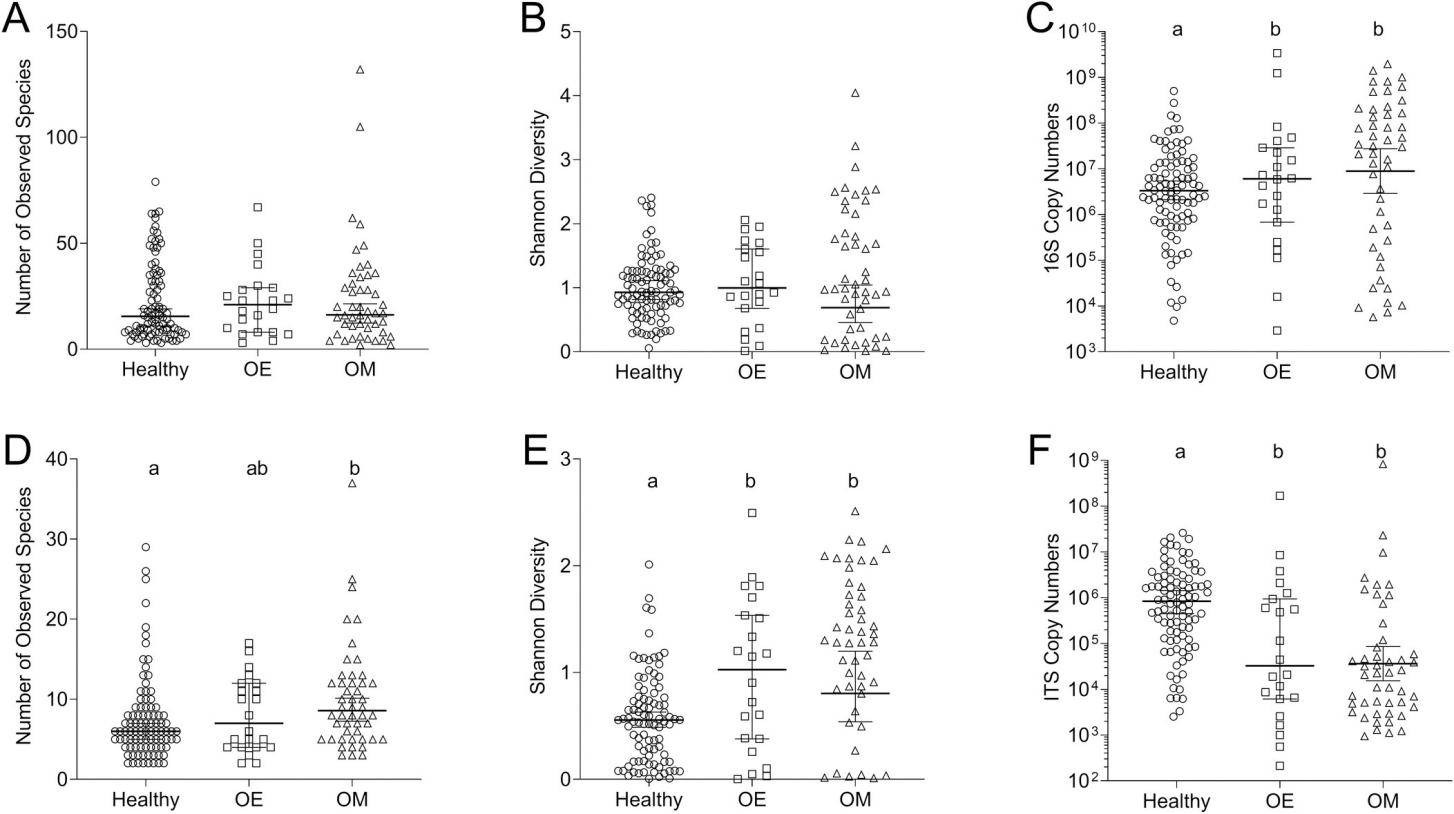
Diversity analysis between groups for bacteria (A-C) and fungi (D-F). Alpha diversity was determined by the number of observed species (A and D) and the Shannon diversity index (B and E). Estimations of the bacterial (C) and fungal (F) biomass were done based on 16S and ITS copy numbers, respectively. Letter (a, b) highlight groups that were significantly different from each other (P > 0.05).

Alpha diversity, which measures the diversity within a given sample (as opposed to beta diversity that measures diversity between samples) showed no significant differences for the bacteriome of the study groups (Fig 2A and 2B, P > 0.05). However, the bacterial biomass based on enumeration of 16S copy numbers revealed a significant increase in bacterial biomass for both OE and OM samples compared to the healthy group (Fig 2C, P = 0.0207).

### Mycobiome composition and diversity

The mycobiome analysis revealed multiple distinct differences between groups: At the phylum level, the mycobiome of healthy ears was dominated by Basidiomycota while in otitis ears Ascomycota was more often detected. At the species level, the most abundant Basidiomycota fungal species detected in the healthy group were from the genus *Malassezia*. Specifically, *Malassezia arunalokei* was the most dominant species (47.32% average abundance, 74/92 samples, Fig 1D), followed by *Malassezia restricta* (35.89%, 90/94), *Malasseziales* sp. (5.95%, 43/94), and *Malassezia globosa* (2.82%, 60/94). Interestingly, three previously reported otopathogens, *Candida metapsilosis/orthopsilosis* (0.43%, 1/94), *Malassezia sympodialis* (0.35%, 24/94), and *Alternaria* sp. (0.33%, 10/94), were detected in the top 15 most abundant species, although at very low abundances (<0.5%) and frequencies.

As was seen in the bacterial analyses, species found in healthy ears were also present in otitis cases, though with lower abundances and frequencies. At the species level, the most abundant Ascomycota fungal species detected in the OE group was *Aspergillus* sp. (23.42%, 8/22), followed by *M*. *restricta* (14.75%, 20/22), *M*. *arunalokei* (12.57%, 16/22), *Candida albicans* (7.68%, 3/22), and *Malasseziales* sp. (5.14%, 10/22) (Fig 1E). In addition to *Aspergillus* sp. and *C*. *albicans*, four other pathogens were among the 15 most abundant species (*Candida parapsilosis* (4.51%, 3/22), *Aspergillus citrinoterreus/pseudoterreus/terreus* (3.71%, 1/22), *Cryptococcus neoformans* (1.84%, 1/22), and *Penicillium* sp. (0.91%, 4/22)). As highlighted by the frequency, single outliers can significantly drive the average species abundances, i.e., *A*. *citrinoterreus/pseudoterreus/terreus* and *C*. *neoformans*, which appeared in only a single sample each.

In the OM group *M*. *restricta* (15.40%, 41/48) was the most abundant species on average, followed by *M*. *arunalokei* (12.97%, 33/48), *Aspergillus* sp. (8.39%, 17/48), and *C*. *parapsilosis* (7.38%, 17/48) (Fig 1F). In addition to *Aspergillus* sp., *C*. *parapsilosis*, and *C*. *metapsilosis*, three other otopathogens were detected among the 15 most abundant species (*Alternaria* sp. (1.97%, 4/48), *C*. *albicans* (1.62%, 2/48), and *Aspergillus flavus* (1.51%, 1/48)). Consistent with the OE group, single outliers in the data significantly drive the group-average species abundances.

There was a significant increase in the number of observed species between the healthy and the OM group (Fig 2D). Shannon diversity showed a significant increase in the number of detected species in both OE and OM compared to the healthy group (Fig 2E). Interestingly, OM and OE groups had a significantly lower fungal biomass as measured by ITS copy numbers compared to the healthy group (Fig 2F). Therefore, a healthy mycobiome was characterized by a lower fungal diversity and a higher fungal biomass compared to clinically affected samples. This is the opposite to what was found for the bacterial biomass.

### Ear microbiome is distinct between healthy and otitis ears

**Fig 3:**
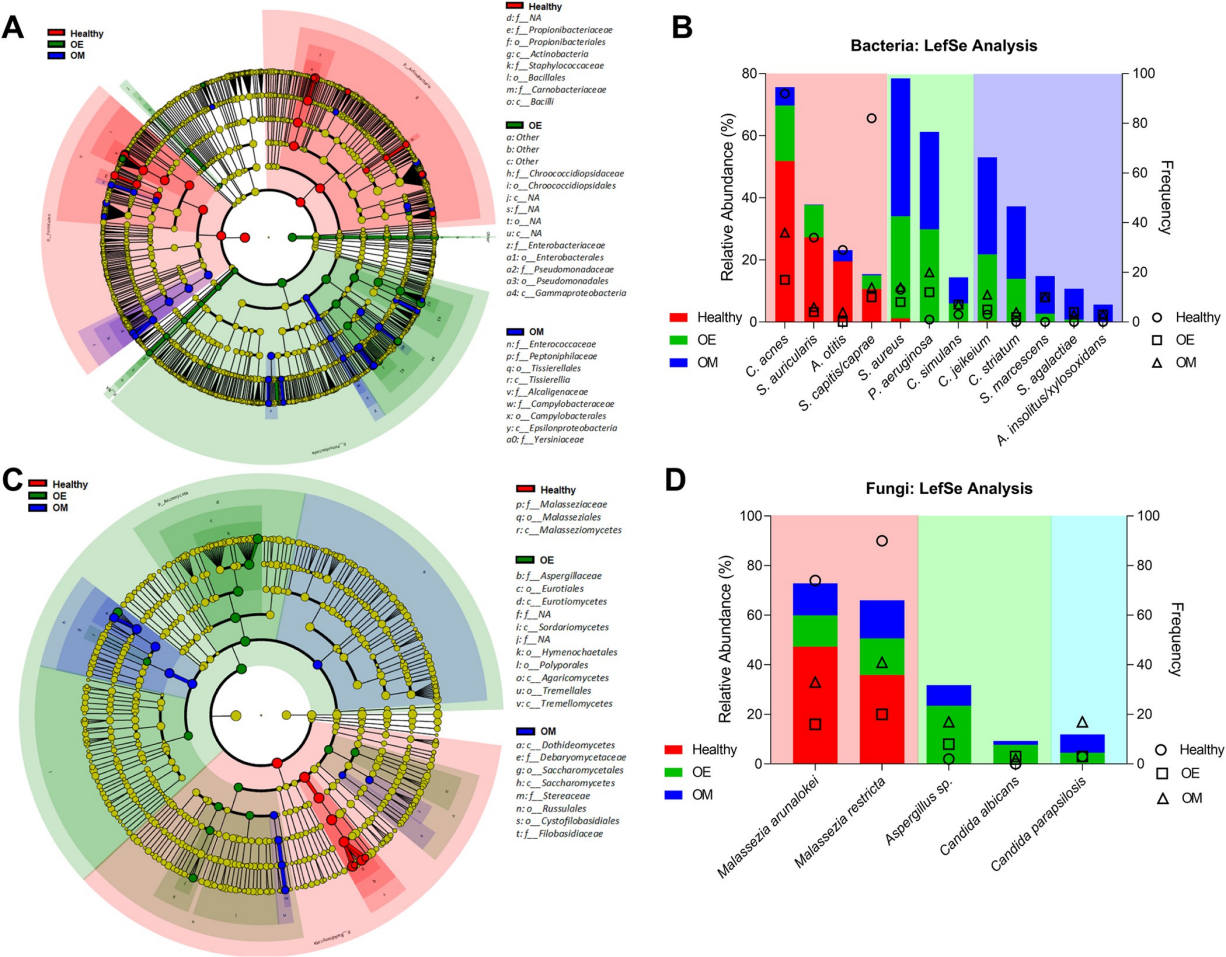
Linear discriminant analysis (LDA) effect size (LEfSe) analysis of otitis microbiota changes due to health context of the patient. Panels highlight taxa significantly different between groups at the phylum to genus level (A, C) and at the species level (B, D) for bacteria (A, B) and fungi (C, D), respectively. Cladograms: Regions in red indicate taxa that are significantly enriched in the healthy group, regions in green represent taxa enriched in the otitis externa group (OE), and regions in blue taxa that are significantly enriched in the otitis media (OM) group. Nods colored in yellow are not significantly different between groups. Each ring represents a taxonomic level, with the phylum level on the outside and the genus level in the innermost ring. Bar graphs: Shown are those species that were significantly different between groups with their relative abundances and frequencies for each group. To visualize the results, the average abundance of each significant species was used for the subset of the samples that had the species present. For readability of the figure, only the species that represented at least 5% of the bacterial or fungal microbiome, respectively are shown.

To identify members of the microbiome that could differentiate the study groups, a LEfSe analysis was conducted at all taxonomic levels. The analysis indicated the phyla Actinobacteria and Firmicutes as significantly enriched in the healthy group, and Proteobacteria in the OE group (Fig 3A). At the species level, 36 significantly distinct bacterial species were identified. For brevity and readability of the graph, only those species that had an abundance of at least 5% in any group are shown (Fig 3B). After applying the 5% filter, four species were significantly enriched in the healthy group, including two commensals, i.e., *C*. *acnes* and *S*. *auriscalis*, and two otopathogens, *A*. *otitis* and *S*. *capitis/caprae*. In the OE group, three species were identified, including the two otopathogens *S*. *aureus* and *P*. *aeruginosa*. OM group was significantly enriched with five previously known otopathogens species, i.e., *C*. *jeikeium*, *C*. *striatum*, *S*. *marcescens*, *Streptococcus agalactiae*, and *Achromobacter xylosoxidans*.

The fungal analysis showed that the phyla Basidiomycota were significantly enriched in the healthy group and Ascomycota in the OE group (Fig 3C). Five species were significantly distinct between groups (using ≤ 5% filter). Specifically, *M*. *arunalokei*, *Malassezia* spp., and *M*. *restricta* were significantly enriched in the healthy group (Fig 3D). Two potentially pathogenic species were significantly enriched in the OE group, *Aspergillus* sp. and *C*. *albicans*. The OM group had a significant enrichment of the otopathogen *C*. *parapsilosis*.

### Ear core microbiome

**Fig 4:**
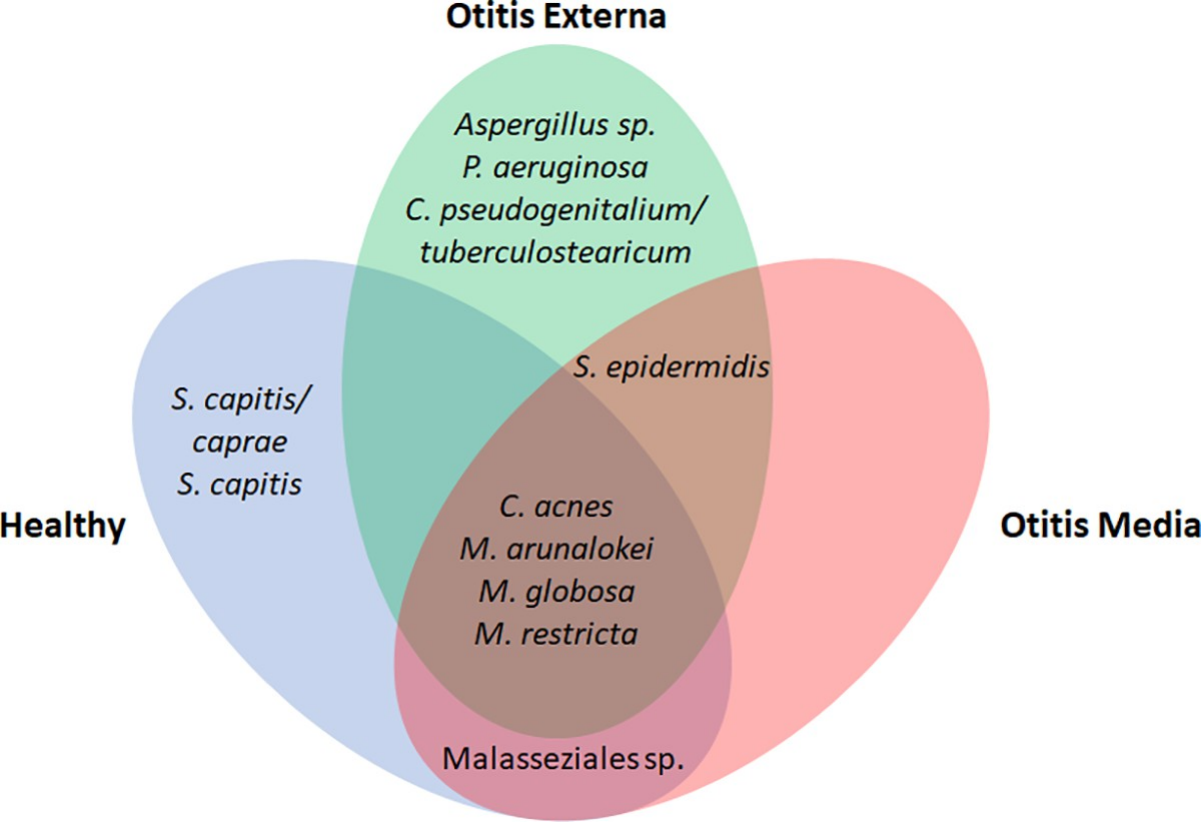
The panels show those species that were part of a shared core microbiome between all three study groups, shared between only two groups, or unique to a given group.

A core microbiome analysis is important for “understanding the stable, consistent components across complex microbial assemblages” and is defined as “the suite of members shared among microbial consortia from similar habitats” [21]. Four species were shared among all groups, *C*. *acnes*, *M*. *arunalokei*, *M*. *globosa*, and *M*. *restricta* (Fig 4). *Staphylococcus epidermidis* was shared between OE and OM. Part of the OE core were *Aspergillus* sp., *P*. *aeruginosa*, and *C*. *pseudogenitalium/tuberculostearicum*, and the healthy group had *S*. *capitis* and *S*. *capitis/caprae* as core members. A taxon from the order Malasseziales was shared only between normal and OM samples.

### Microbial composition clustering

**Fig 5:**
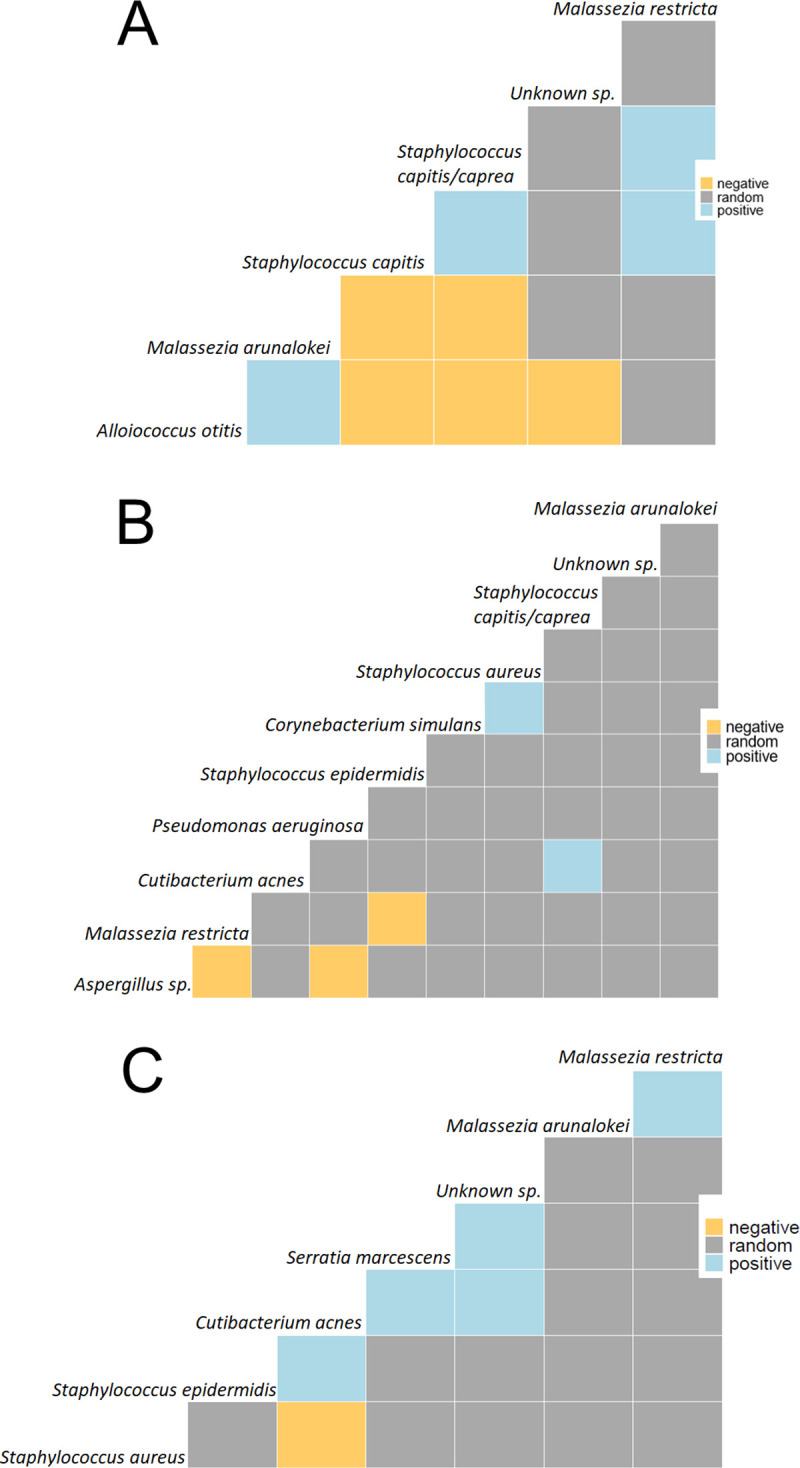
Co-occurrence analysis. Microbial interactions between bacterial and fungal species in each study group. Negative interactions between species are shown in yellow, positive interactions are shown in blue and no significant interaction is shown in grey. (A) healthy, (B) otitis externa, and (C) otitis media.

Co-occurrence analysis, which highlights interspecies dependencies based on patient health context (Fig 5), showed that interspecies dependencies were health context and species specific. In the healthy group, *S*. *capitis* and *S*. *capitis/caprae* showed a positive co-occurrence with each other, and negative interactions with *M*. *arunalokei* and *A*. *otitis*, which was only seen in that group (Fig 5A). The OE group showed two positive interactions, one between *C*. *acnes* and *S*. *capitis/caprae*, and one between *S*. *aureus* and *C*. *simulans* (Fig 5B). A negative co-occurrence between *M*. *restricta* and the otopathogen *Aspergillus* sp. was detected, hinting at a protective effect of *Malassezia* species. Two pathogens from different kingdoms, *Aspergillus* sp. and *P*. *aeruginosa*, had a negative interaction. Interestingly, the OM group was characterized by a positive co-occurrence between *S*. *marcescens* and *C*. *acnes*. *C*. *acnes* further had a negative interaction with *S*. *aureus*. The OM samples were characterized by positive co-occurrences between ear commensals i.e., *Malassezia* species, and *C*. *acnes* and *S*. *epidermidis* (Fig 5C). The otopathogen *S*. *aureus* had a negative co-occurrence with the commensal *C*. *acnes*.

### NGS reveals pathogen dysbiosis by bacterial, fungal, or joint players in otitis patients

Several individual samples were characterized by a very low species diversity, i.e., a single species represented > 90% of either the bacteriome (n = 29) or mycobiome (n = 35). Based on the previous observation that the microbial profile is highly individualized for OE and OM patients (Fig 1B, 1C, 1E and 1F), the bacterial and fungal profiles for each OM and OE patient were analyzed to identify potential pathogens that may be driving the infection in each case. A total of 18% (4/22) of OE patients showed a single bacterial species representing ≥80% of the total bacteriome, and 32% (7/22) of patients had a single fungal species at ≥80% of the total mycobiome. Dual bacterial and fungal pathogen dysbiosis was seen in 9% (2/22) of patients (Table 2). For OM patients, overrepresentation of a single bacterial pathogen was more common with 35% (17/48) of patients, while 15% (7/48) showed a single fungal pathogen overrepresentation (Table 3). There was no fungal otopathogen overrepresentation (≥90%) seen in the healthy group and only one sample showed a bacterial pathogen at 92% (*A*. *otitis*) (Fig 1A and 1D). Overall, 38% (11/92) of healthy group samples had a single bacterium, *C*. *acnes*, represent ≥90% of the bacteriome. A possible assumption when such a dysbiosis is detected, is that there may be an overgrowth, i.e., increase of microbial biomass, in the sample. A correlation analysis between the number of observed species and the biomass, as estimated by 16S and ITS copy numbers, however, showed no significant correlation (Bacteria: r = -0.042, P = 0.595; Fungi: r = 0.110, P = 0.162, S4 Fig).

**Table 2:** Pathogens detected in otitis externa (OE) patients at high relative abundances.

Patient	Gender	Otopathogens detected by NGS test (% Rel. Abund.)	Pathogen Type
1	M	*S*. *aureus* (68%)	Bacterial
2	M	*S*. *aureus* (80%)	Bacterial
3	F	*Aspergillus piperis* (99%)	Fungal
4	F	*Aspergillus piperis* (100%)	Fungal
5	F	*S*. *aureus* (85%); *Candida albicans* (80%)	Bacterial &Fungal
6	M	*Aspergillus* sp. (94%)	Fungal
7	M	*Pseudomonas aeruginosa* (98%)	Bacterial
8	M	*Pseudomonas aeruginosa* (71%)	Bacterial
9	F	*Aspergillus terreus* (82%)	Fungal
10	M	*Klebsiella pneumoniae* (94%)	Bacterial
11	M	*Candida parapsiliosis* (88%)	Fungal
12	F	*Proteus Mirabilis* (81%); *Aspergillus* (100%)	Bacterial & Fungal
13	F	*Corynebacterium freneyi* (93%)	Bacterial
14	M	*Pseudomonas aeruginosa* (100%)	Bacterial
15	F	*Serratia marcescens* (42%); *P*. *melaninogenica* (40%); *C*. *albicans* (90%)	Bacterial &Fungal
16	F	*Aspergillus* sp. (89%)	Fungal

Abbreviations: Rel.: Relative; Abund.: Abundancy, M: Male; F: Female.

**Table 3:** Pathogens detected in otitis media (OM) patients at high relative abundances.

Patient	Gender	Pathogens ID by NGS test (% Rel. Abund.)	Pathogen Type
1	M	*Pseudomonas aeruginosa* (99%)	Bacterial
2	F	*Pseudomonas aeruginosa* (97.4%)	Bacterial
3	F	*Corynebacterium freneyi* (91.4%)	Bacterial
4	M	*Aspergillus piperis* (100%)	Fungal
5	F	*S*. *aureus* (97%); *Aspergilllus terreus* (100%)	Bacterial & Fungal
6	M	*S*. *aureus* (100%)	Bacterial
7	M	*Pseudomonas aeruginosa* (100%)	Bacterial
8	F	*Candida metapsilosis* (78%)	Fungal
9	F	*Proteus mirabilis* (65%); *Aspergillus* sp. (96%)	Bacterial & Fungal
10	M	*P*. *aeruginosa* (56%); *Brevibacterium otitidis* (38%)/ *Candida parapsilosis* (76%)	Bacterial & Fungal
11	M	*Aspergillus flavus* (73%)	Fungal
12	F	*Weeksella virosa* (51%); *Pseudoclavibacter bifida* (19%); *Oligella urethralis* (15%)	Bacterial
13	F	*Pseudomonas aeruginosa* (84%)	Bacterial
14	F	*Aspergillus piperis* (80%)	Fungal
15	F	*Aspergillus piperis* (100%)	Fungal
16	F	*P*. *aeruginosa* (81%)	Bacterial
17	M	*S*. *aureus* (88%)	Bacterial
18	F	*Candida metapsilosis* (73%)	Fungal
19	M	*S*. *aureus* (99.5%)	Bacterial
20	M	*S*. *aureus* (99%)	Bacterial
21	F	*Moraxella catarrhalis* (94%)	Bacterial
22	M	*S*. *aureus* (97%)	Bacterial
23	F	*Candida parapsilosis* (99%)	Fungal
24	F	*Serratia marcescens* (99%)	Bacterial
25	M	*Malassezia sloofiae* (86%)	Fungal
26	F	*Corynebacterium resistens* (91%)	Bacterial
27	F	*Candida metapsilosis* (100%)	Fungal
28	F	*C*. *jeikeium* (71%)	Bacterial
29	F	*C*. *jeikeium* (80.5%)	Bacterial
30	M	*Pseudomonas aeruginosa* (100%)	Bacterial
31	M	*Auritidibacter* sp. (100%)	Bacterial

Abbreviations: Rel.: Relative; Abund.: Abundancy, M: Male; F: Female.

**Fig 6:**
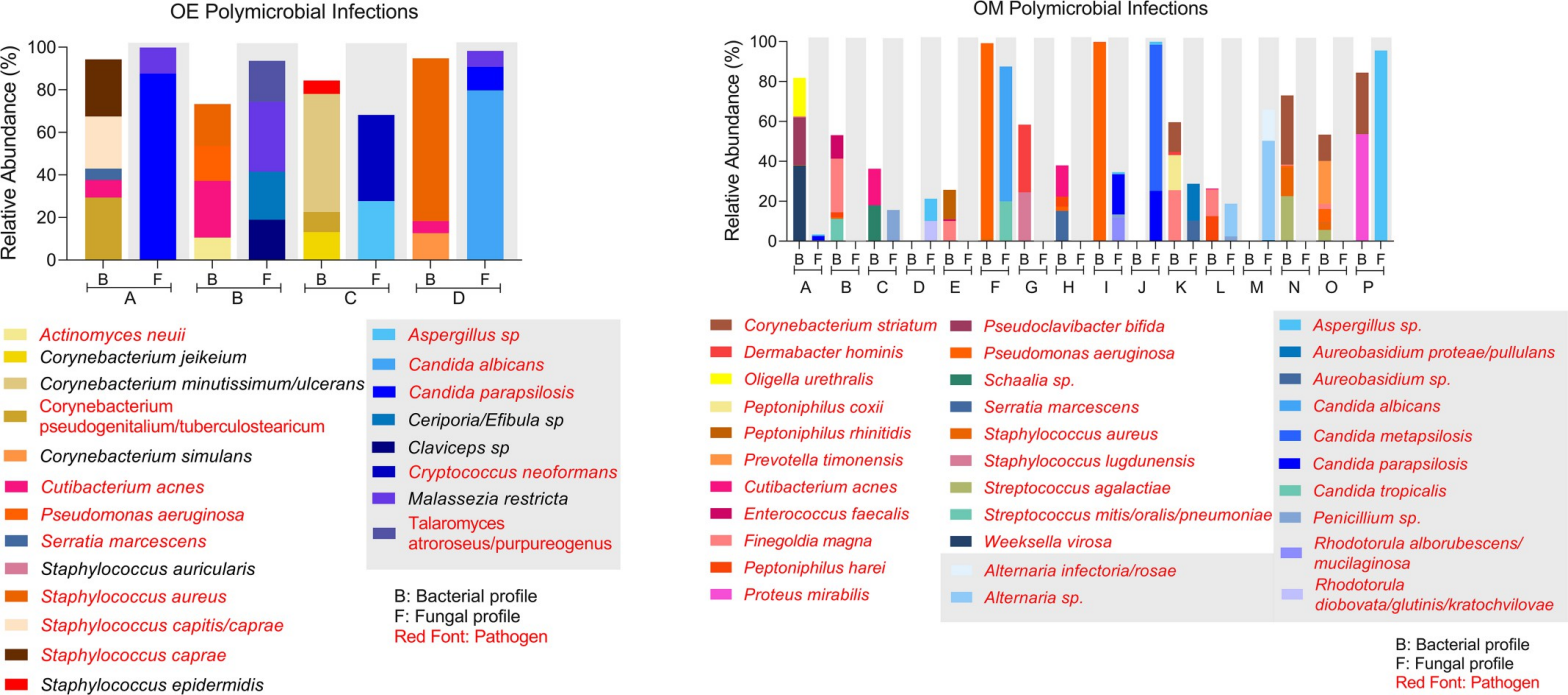
Poly-species infection analysis. As an example, and for brevity of the graph, the bacterial and fungal profiles of those patients that were positive for at least three bacterial otopathogens or two fungal otopathogens are shown for (A) OE and (B) OM.

Polymicrobial infections are a concern for OE and OM, which may be either multiple bacterial or fungal pathogens or a combination. OE and OM samples were analyzed for the presence of multiple pathogens in each sample with a cutoff of ≥10% relative abundance in the bacteriome or mycobiome. All OE patients had at least one bacterial otopathogen present at ≥10% of the bacteriome, 36% (8/22) had two (≥10% each), one patient had three (Fig 6A, sample A), and one patient showed four otopathogens present at ≥10% (Fig 6A, sample B). Between the two patients that showed three and four pathogens respectively, there was no overlap in the pathogens detected. The mycobiome in the OE group showed that 45% (10/22) patients had one and 9% (2/22) patient had two fungal pathogens (Fig 6A).

In the OM group, 40% (19/48) of patients had one bacterial pathogen present at ≥10%, 21% (10/48) had two and for one patient three otopathogens were detected (Fig 6B). In the mycobiome, 40% (19/48) of patients had one and six individuals had two fungal otopathogens present at ≥10% (13%). About 35% (17/48) of patients had both a bacterial and a fungal pathogen present at ≥10% (Fig 6B). As seen for OE and OM polymicrobial analysis, the combination of bacterial and/or fungal pathogens is highly individualized in this data set.

## Discussion

Acute and chronic adult ear infections are common, but their etiology and the role of the eukaryotic mycobiome remain inadequately investigated. We investigated microbial community profiles in healthy adult and OM and OE patient ears, and all harbored both bacteria and fungi. In the healthy ear, the most abundant bacterial species were *C*. *acnes*, *S*. *auriscularis*, and *S*. *captis/caprae*, consistent with previous investigations [22]. The healthy mycobiome was characterized by high numbers of *Malassezia* with two distinct groups harboring either *M*. *arunalokei* or *M*. *restricta*. While *M*. *restricta* is well characterized and generally regarded as a commensal on normal skin with no reports of it being a human otopathogen, it can be associated with skin diseases like dandruff [23]. *M*. *arunalokei* has only recently been identified [24] and remains minimally investigated [25]. Further investigation into the role of these species in healthy ears is warranted. In previous NGS studies *M*. *arunalokei* would have appeared as “*Malassezia* spp.” or in unidentified sequence “dark matter”.

Compared to OE and OM, the commensals *C*. *acnes* and *S*. *capitis/caprae* were significantly enriched in the healthy group, which confirms previous reports, as well as *M*. *arunalokei* and *M*. *restricta*. The otopathogens *S*. *auricularis* [10] and *A*. *otitidis* [11] were also significantly enriched in this group, which was unexpected and highlights the need for continual refinement of research databases and careful consideration of unidentified species. An unexpected frequency of previously postulated otopathogens i.e., *C*. *otitidis* or *S*. *caprae*, in the healthy ear was detected, however their frequencies and abundances were increased in OE and OM groups. These findings are most likely due to the increased sensitivity of the 16S pipeline [19] versus earlier culture-based methods and the potential lack of testing on clinically healthy ears. This highlights the need for continual refinement of research databases and careful consideration of unidentified species.

In OE the most abundant species were *P*. *aeruginosa*, *C*. *acnes*, and *S*. *aureus* with eight otopathogens among the top 15 most abundant; and in OM by *P*. *aeruginosa*, *S*. *aureus*, and *C*. *jeikeium*, with ten otopathogens among the top 15. The commonality of *P*. *aeruginosa* and *S*. *aureus* as dominant pathogens in the bacterial analyses of both OM and OE supports previous findings and validates the NGS study results. Previous studies had identified *Turicella otitidis*, *Alloiococcus otitidis*, and *Staphylococcus auricularis* as primary otopathogens, which were present in OM cases at lower abundances [10]. Interestingly, this study only reported OM cases and did not compare results with a healthy age-matched cohort. From our results, these organisms are part of the normal ear flora. This shows that molecular testing has the potential to provide more detailed information about the bacterial etiology of AOM.

The mycobiome was surprisingly distinct between groups. In both OE and OM, *Malassezia* spp. remained highly abundant mycobiome members, but newly appearing non-*Malassezia* species were increased in abundance. In OE the two most abundant non-*Malassezia* species were *Aspergillus* sp., followed by *C*. *albicans*, and *Aspergillus* spp. in the OM group (17/48 samples). *Aspergillus* sp. [26] and *C*. *albicans* [27], potential pathogens, were significantly enriched in OE, and OM was enriched for the previously characterized otopathogen *C*. *parapsilosis* [28, 29]. As highlighted by the dysbiosis and overgrowth analysis of individual OE and OM samples, the microbial composition was highly personalized. Therefore, averaging relative abundance across all OM samples may dilute the contribution of kingdom-specific pathogens. This issue may be resolved by analyzing a larger sample size, which may confirm the findings presented here that the microbiome of OE and OM patients is highly individualized. The high degree of inter-subject variances provides a challenge to building microbiome models/profiles associated with each disease state.

High level beta-diversity analysis indicated sample clustering for bacteria but none for fungi. However, the mycobiome alpha-diversity was significantly altered: the number of detected fungal species was significantly higher in the OM group compared to healthy samples, and the Shannon diversity was significantly higher in both otitis groups compared to healthy samples. Thus, healthy ears were characterized by a lower fungal diversity, dominated by two *Malassezia* species, i.e., *M*. *restricta* and *M*. *arunalokei*, and higher fungal biomass compared to infected ears. Comparison of subjects with limited microbial diversity (dysbiosis) showed no correlation to healthy or diseased ears, indicating disease is unlikely directly linked to bacterial overgrowth but may indicate a mutualistic fungal/host relationship, where loss of a protective fungal mutualist correlates with the increase in bacterial biomass. Biomass was estimated as bacterial and fungal cell numbers using 16S rDNA and ITS analysis by enumerating copy numbers [30]. While complicated by diversity in fungal ITS copy number, the ITS copy number may also serve as a proxy for fungal cell number [19]. These enumerations showed a significant increase in bacteria and a decrease in fungi for both OE and OM compared to the healthy group. It is important to note that while the 16S bacterial copy numbers were higher than the fungal ITS copy numbers for all groups, it must be considered that due to their significantly larger size (9–12μm versus 0.8–1μm diameter) and the cubic relationship between diameter and mass, each fungal genome represents greater than 1000x more active biomass. Further longitudinal studies and development of more accurate estimates of fungal ITS copy number per cell may help untangle the inter-kingdom relationships [31].

Interkingdom interactions are complex and difficult to decipher via presence/absence analyses, but co-occurrence analysis highlights potential interspecies interaction. These interactions were species and health state dependent. In healthy ears *S*. *capitis* and *S*. *capitis/caprae* showed a positive co-occurrence, but interestingly negative interactions with the healthy ear-associated *M*. *arunalokei*. The OE group showed positive interactions between *C*. *acnes* and *S*. *capitis/caprae*, and between *S*. *aureus* and *C*. *simulans*. *C*. *simulans* is known to cause skin and soft tissue infections [32] but has not previously been identified as an ear pathogen. Based on the data presented here, *C*. *simulans* could potentially represent a new biomarker for OE infections. The negative co-occurrence between *M*. *restricta* and the known otopathogen *Aspergillus* was detected, potentially indicating mutualistic benefit of *Malassezia*. *Malassezia* have the potential to be commensal (as they are found on all human skin [25]), pathogenic (as in seborrheic dermatitis [33] and Crohn’s disease [34]), or mutualistic (as in some cases of atopic dermatitis) [35]. In this study *Malassezia*, particularly *M*. *arunalokei* and *M*. *restricta*, appear to most likely be mutualists. Inter-kingdom antagonistic effects were seen between two pathogens from different kingdoms, *Aspergillus* sp. and *P*. *aeruginosa*, but in one case, OM, there was a positive co-occurrence between *S*. *marcescens* and *C*. *acnes*. It is possible this represents strain specificity in *C*. *acnes*, as strains can be either pathogenic or commensal [31]. In the case of OM there were positive interactions between the commensals *Malassezia* species and *C*. *acnes* and *S*. *epidermidis*, while the otopathogen *S*. *aureus* had a negative co-occurrence with the commensal *C*. *acnes*. Specific implications of this finding for diagnostic and treatment purposes remain to be investigated.

The core microbiome represents the stable, consistent components of a microbial community. The shared human ear core microbiome consisted of prokaryote *C*. *acnes* and eukaryotic *M*. *arunalokei*, *M*. *globosa*, and *M*. *restricta*, which confirms the potential mutualistic effect of *Malassezia* species as discussed above.

This detailed ear microbiome NGS analyses revealed new, more complex relationships between microbial community members. Inclusion of the fungal kingdom disclosed significant changes in mycobiome diversity and biomass between healthy and diseased ears, and reveals the presence of a potential mutualistic, protective effect of *Malassezia* species.

## Conclusions

The human ear microbiome remains inadequately investigated and further investigation will be important in definition of ear health and treatment of ear disease. Using NGS as a testing tool could improve treatment outcome, guide the selection of appropriate therapy, and limit inappropriate antibacterial treatments and thus improve global antimicrobial stewardship. This study highlighted the importance of pathogen classification on a per case basis.

## Supporting information

S1 Fig Microbial stability in DNA/RNA Shield. ZymoBIOMICS Microbial Community Standard (Zymo Research Corp.) microbial profiles. This standard is composed of 8 bacterial and 2 fungal species in DNA/RNA Shield (Zymo Research Corp.). A and B show microbial profiles at different time points at ambient temperature. Each time point was done in duplicate, and samples were taken at days 1, 4, 7, 14, and 30. A. Microbial composition barplots showing relative abundance of 8 bacterial species, Pa (*Pseudomonas aeruginosa*), Se (*Salmonella enterica*), Ec (*Escherichia coli*), Lf (*Lactobacillus fermentum*), Ef (*Enterococcus faecalis*), Sa (*Staphylococcus aureus*), Lm (*Listeria monocytogenes*), and Bs (*Bacillus subtilis*) over time. B. Microbial composition barplots showing relative abundance of 2 fungal species, Cn (*Cryptococcusneoformans*), Sc (*Saccharomyces cerevisiae*) over time.
(JPG)

S2 Fig Bray-Curtis Beta diversity analysis for bacteria (A) and fungi (B). Healthy samples are shown in red, otitis externa samples in green, and otitis media samples in blue.
(PNG)

S3 Fig Bray-Curtis Beta diversity analysis for bacteria based on ear side of the same individual. Left ears samples are shown in red, right ear samples in blue.
(PNG)

S4 Fig Correlation analysis between the number of observed species (x axis) and the estimated absolute numbers for (A) bacteria and (B) fungi.
(PDF)

S1 Table Relative abundance table for bacterial profile per sample. (TXT)

S2 Table Relative abundance table for fungal profile per sample. (TXT)
