# Supplementary figures and images for: The adult microbiome of healthy and otitis patients: Definition of the core healthy and diseased ear microbiomes
Source: PLoS One. 2022 Jan 24;17(1):e0262806. doi: 10.1371/journal.pone.0262806 (PMC8786117; doi:10.1371/journal.pone.0262806)

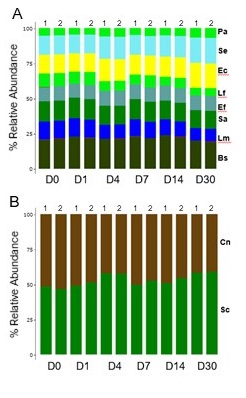

Supplement: S1 Fig — ZymoBIOMICS Microbial Community Standard (Zymo Research Corp.) microbial profiles. This standard is composed of 8 bacterial and 2 fungal species in DNA/RNA Shield (Zymo Research Corp.). A and B show microbial profiles at different time points at ambient temperature. Each time point was done in duplicate, and samples were taken at days 1, 4, 7, 14, and 30. A. Microbial composition barplots showing relative abundance of 8 bacterial species, Pa (Pseudomonas aeruginosa), Se (Salmonella enterica), Ec (Escherichia coli), Lf (Lactobacillus fermentum), Ef (Enterococcus faecalis), Sa (Staphylococcus aureus), Lm (Listeria monocytogenes), and Bs (Bacillus subtilis) over time. B. Microbial composition barplots showing relative abundance of 2 fungal species, Cn (Cryptococcusneoformans), Sc (Saccharomyces cerevisiae) over time. (JPG) [file pone.0262806.s001.jpg]

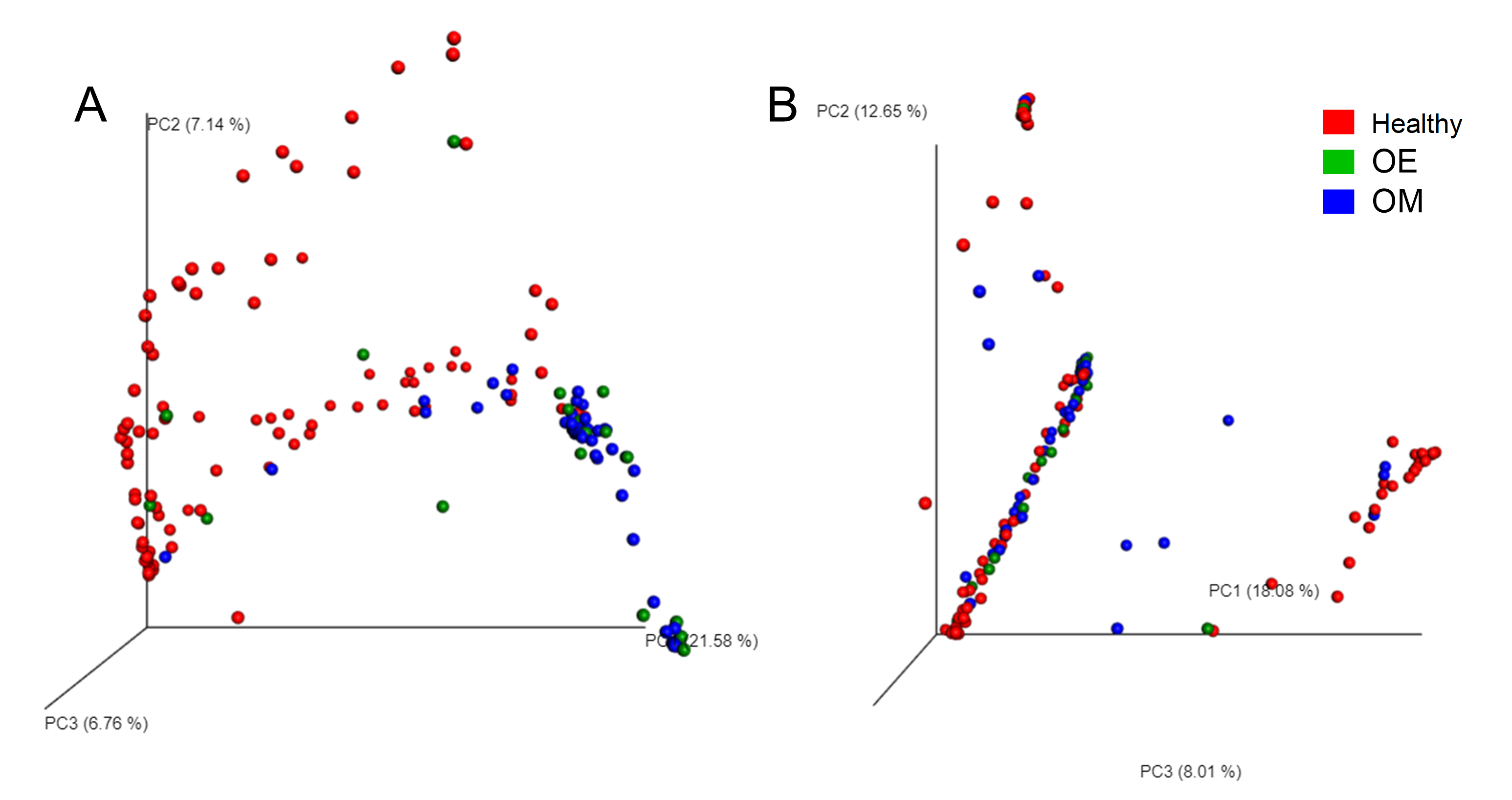

Supplement: S2 Fig — Bray-Curtis Beta diversity analysis for bacteria (A) and fungi (B). Healthy samples are shown in red, otitis externa samples in green, and otitis media samples in blue. (PNG) [file pone.0262806.s002.png]

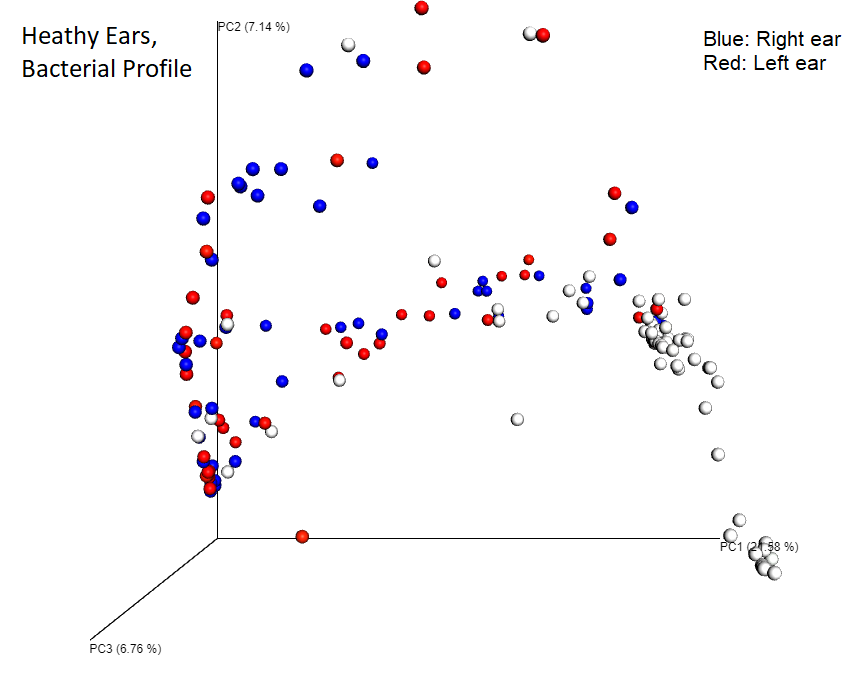

Supplement: S3 Fig — Left ears samples are shown in red, right ear samples in blue. (PNG) [file pone.0262806.s003.png]

**A**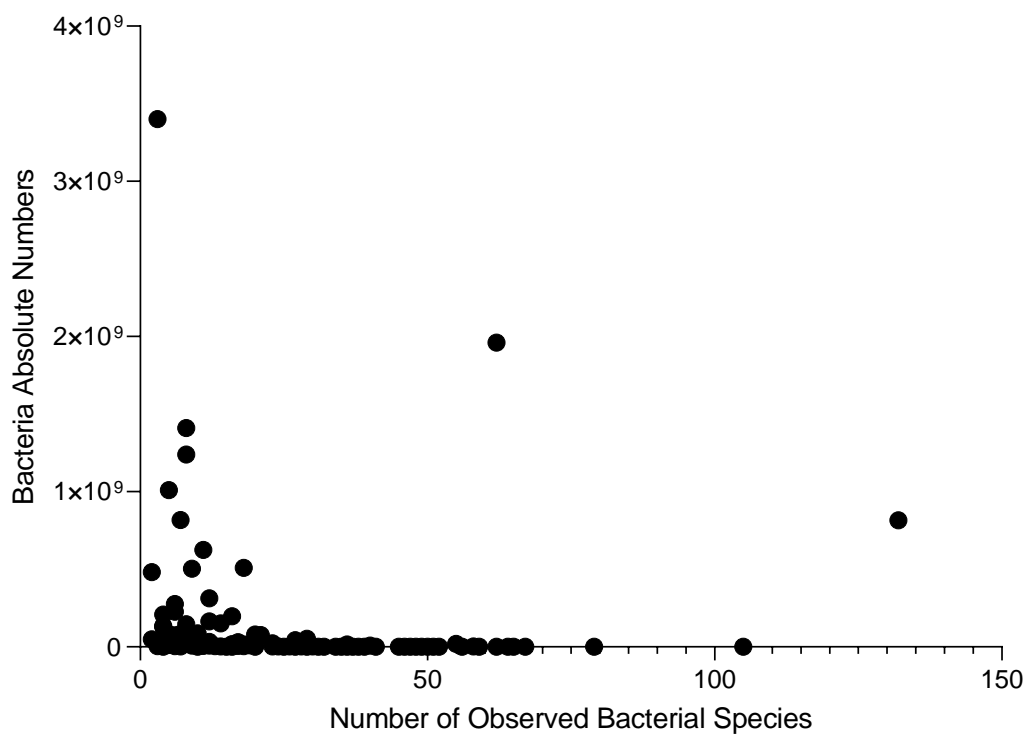**B**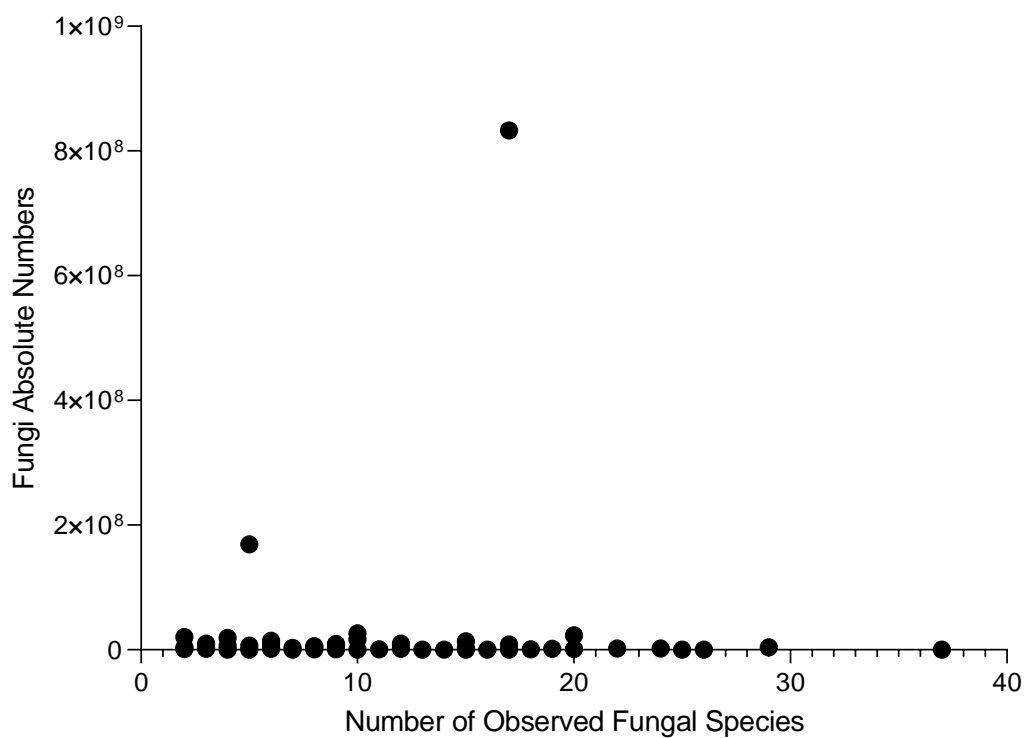

Supplement: S4 Fig — Correlation analysis between the number of observed species (x axis) and the estimated absolute numbers for (A) bacteria and (B) fungi. (PDF) [file pone.0262806.s004.pdf]
